# Glycolysis regulates palatal mesenchyme proliferation through* Pten-Glut1* axis via* Pten* classical and non-classical pathways

**DOI:** 10.1007/s10565-025-10000-2

**Published:** 2025-02-27

**Authors:** Yijia Wang, Xia Peng, Xiaotong Wang, Jing Chen, Xiaoyu Zheng, Xige Zhao, Cui Guo, Juan Du

**Affiliations:** 1 Laboratory of Orofacial Development, Laboratory of Molecular Signaling and Stem Cells Therapy, Molecular Laboratory for Gene Therapy and Tooth Regeneration, Beijing Key Laboratory of Tooth Regeneration and Function Reconstruction, Capital Medical University School of Stomatology, No.9 Fanjiacun Road, Beijing, 100070 China; 2 Laboratory of Tissue Regeneration and Immunology and Department of Periodontics, Beijing Key Laboratory of Tooth Regeneration and Function Reconstruction, Capital Medical University School of Stomatology, No.9 Fanjiacun Road, Beijing, 100070 China; 3 Department of Geriatric Dentistry, Capital Medical University School of Stomatology, Fanjiacun Road No.9, Beijing, 100070 China

**Keywords:** Pten, Cleft palate, Interceptive treatment, Proliferation, Glycolysis

## Abstract

Abnormal embryonic development leads to the formation of cleft palate (CP) which is difficult to be detected by genetic screening and needs sequent treatment from infants to adults. There are no interceptive treatment about CP until now. Germline deletion of phosphatase and tensin homolog (*Pten*) was related to embryonic malformation and regulated tumor cell proliferation through glycolysis. However, the role of *Pten* in CP and the relationship between CP, *Pten,* and glycolysis are unknown. In our research, we constructed *Pten* knockdown models in vitro and in vivo. Our results provided preliminary evidence that blocking *Pten* by its inhibitor such as VO-OHpic might be an effective interceptive treatment in early period of palate development when pregnant mother expose in harmful environment during the early period of palate development to reducing CP occurring which was related with the crosstalk between *Pten*, and glycolysis in the process.

**Graphical Abstract:**

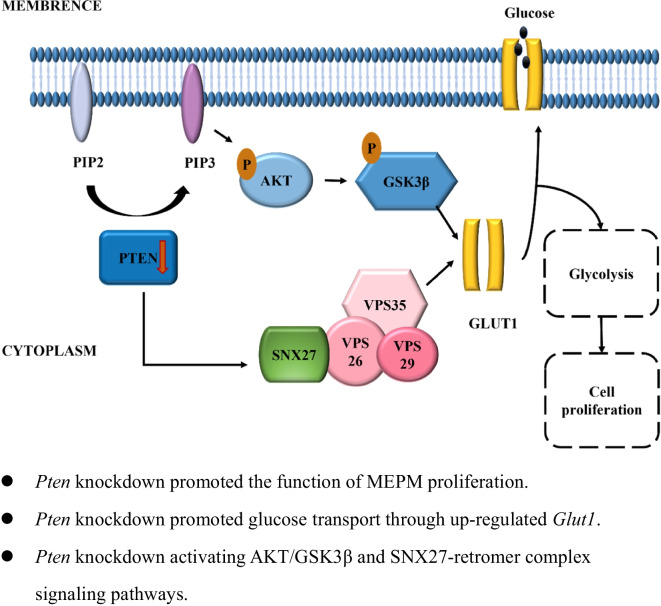

**Supplementary Information:**

The online version contains supplementary material available at 10.1007/s10565-025-10000-2.

## Introduction

Recent studies have found that birth defects account for 8% and 10% of deaths among children under 5 and newborns globally, respectively (Strong et al. 2024). Birth defects are an important cause of neonatal death (Matthews et al. 2015). Among them, cleft palate (CP) is one of the most common congenital craniofacial malformation, which disrupts the soft and hard tissues around the cavity of the mouth throughout the process of embryonic development. The etiology of CP is the interaction of environmental (smoking, drinking, drugs, etc.) and genetic factors (some cell signalings such as SHH, TGFß, etc.) (Behnan et al. 2005; Lewis et al. 2017; Moreau et al. 2007). At present, there are no genetic screening and interceptive treatment to reducing the occurrence of CP. The way of repairing CP is mainly postnatal surgical treatment (Chepla and Gosain 2013). However, the treatment of CP extends from infancy through adolescence. Over the years, the children and their families have struggled with multiple surgeries, increasing the financial burden and seriously impacting the child's psychological well-being (Kapp-Simon 2004). Therefore, it is particularly urgent to study the pathogenesis of CP, especially gene-related pathogenesis to find genetic screening or interceptive treatment to reduce its incidence.

The deletion of the phosphatase and tensin homolog (*Pten*) gene in the germline of mice has been shown to cause embryonic abnormalities, as *Pten* is a crucial gene involved in regulating growth and survival (Di Cristofano et al. 1998). *Pten* controls various cellular functions such as cell survival, proliferation, energy metabolism, and cellular architecture by dephosphorylating phosphatidylinositol-3,4,5-trisphosphate (PIP3) (Dahia 2000; Song et al. 2012). PIP3 is an activator of the 3-phosphoinositide-dependent kinase (PDK) and Akt/protein kinase B (PKB) (Fruman et al. 1998). Loss of *Pten* function could increase levels of PIP3 and active phosphoinositide 3-kinase (PI3K)-AKT pathway (Maehama and Dixon 1998; Qian et al. 2019). Current studies have shown that *Pten* was essential for craniofacial morphogenesis. Clinical and molecular data have revealed that macrocephaly with development was seen in *Pten* mutation (Hansen-Kiss et al. 2017). And *Pten* knockout mice also displayed malformation of craniofacial structures such as overgrowth of craniofacial structures, increasing cell proliferation rate and enhancing osteoblast differentiation (Yang et al. 2018).

All of these studies have confirmed that *Pten* plays a major part in cell proliferation and craniofacial development. However, the specific mechanism by which it contributes to palate development remains unclear. Interference with palate shelves elevation, attachment, or fusion finally causes CP. In this process, the proliferation of mesenchymal cells acts a crucial role in the formation of the palate shelf (Liu et al. 2021; Okello et al. 2017). Retinoic acid (RA) inducing CP is one of the most commonly used CP models, primarily by inhibiting the proliferative capacity of the palatal mesenchyme to induce CP (Nugent et al. 1998). Here, in vivo, we firstly set up a *Pten* knockdown model by intraperitoneal injection of the *Pten* inhibitor VO-OHpic to RA-induced pregnant mice according to the enhanced proliferation derived from *Pten* knockdown. Our results found that *Pten* knockdown could rescue CP which caused by RA, but the underneath mechanism was not clear. According to that, we further knocked down *Pten *in vitro to investigate its specific effect on mouse embryonic palatal mesenchymal cells. Our objective was to identify a novel target for protective or interceptive treatment to lessen the occurrence of CP as well as to understand the molecular mechanism of *Pten* during palate development both in vitro and in vivo.

## Materials and methods

### Construction of Pten low expression rescue model

Pregnant ICR mice and C57BL/6 mice were bought from Sibeifu Company (Beijing, China). Healthy C57BL/6 female mice (6–8 weeks, 22–25 g) were selected and caged 2:1 with healthy males (8–10 weeks) overnight, and the vaginal plugs were examined on the next morning and recorded as embryonic day (E) 0.5. At E10.5, mice in the RA group were on gavage with retinoic acid (RA, 100 mg/kg, R2625, Sigma-Aldrich, Germany), and the control group was on gavage with an equal amount of corn oil. *Pten* inhibitor VO-OHpic (100 μg/kg, HY-110067, MedChemExpress, China) was injected intraperitoneally at E10.5–13.5 as the RA + VO-OHpic group. At E13.5 or E16.5, pregnant mice were executed by cervical dislocation and the craniofacial of fetus mice were carefully extracted. A portion of heads was embedded for cranial coronal sectioning, while some of the palatal tissues were taken for further investigations. The Animal Care and Use Committee of the Capital Medical University School of Stomatology (permission number: KQYY-202109–006) approved all operations involving mice, and all studies were conducted in accordance with applicable regulations. The ARRIVE rules were adhered to in all animal trials. The suffering of animals was reduced.

### Hematoxylin–eosin (H&E) staining

The E13.5 and E16.5 palate tissue slices (5 μm thick) were prepared. For 5 min each time, xylene was used to remove the wax from the paraffin pieces. They were then rehydrated in a gradient of alcohol. The pieces were rinsed with flowing water and thereafter treated with hematoxylin for a period of 1 min. After the previous procedures, eosin was used to stain the pieces for 2 min and the pieces were dried out with a gradient alcohol. Then clean xylene was applied to eliminate impurities.

### Immunohistochemistry (IHC)

The same steps were used to prepare the slices for IHC as for H&E. It was heated to 65 ℃ for 1 h to remove the paraffin, and then the pieces were rehydrated. After being treated with antigen retrieval, the slices were followed by immersing in 10% H_2_O_2_/methanol in order to halt the peroxidase activity. Following this, the slices were incubated with goat serum (ZLI-9021, ZSGB-BIO, China) in order to halt the binding of non-specific antibodies. After incubated the first and second antibody (Table S1), the slides were coated with DAB (D4293, Sigma-Aldrich, Germany). The regions of interest were dyed with a brown to yellow color. The relative expression level was measured using ImageJ (1.48v).

### Isolation and culture of mouse embryonic palatal mesenchymal (MEPM) cells and lentiviral transfection of Pten

To obtain MEPM cells, we adopted the one-step method, the same as our lab previously (Wang et al. 2022). The following experiments were performed on the cells of the passage (P) 2–4 MEPM cells. In order to attain the most effective silencing of the *Pten* gene, three distinct small-hairpin sequences (1689, 1793, 1927, Table S2) were specifically constructed for targeting *Pten*. After infected, MEPM cells were screened by 1.5 μg/mL puromycin (A1113803, ThermoFisher, United States). RNA and protein were collected and the most beneficial sequence was chosen.

### Total RNA extraction and quantitative real-time polymerase chain reaction (qRT-PCR)

Total RNA was extracted by Trizol (CW0580, ComWin Biotech, China) and cDNA was obtained by Plus All-in-one 1st Strand cDNA Synthesis SuperMix (E047, Novoprotein, China). In Table S3, the primer sequences for qRT-PCR were presented. The final data were standardized to the levels of β-actin and examined by computing the comparative cycle threshold values (2^−ΔΔCt^).

### Western blot

The protein of MEPM cells was extracted by radioimmunoprecipitation assay buffer (RIPA buffer, C1053, Applygen, China) that contained phenylmethylsulfonyl fluoride (PMSF, 93482, Sigma-Aldrich, Germany) and protease inhibitor cocktail (PIC, P8340, Sigma-Aldrich, Germany). Then the protein was moved onto polyvinylidene fluoride (PVDF) membranes (IPVH00010, Millipore, United States) and incubated with the primary and secondary antibodies (Table S1). Target protein bands were quantified by ImageJ.

### *5-Ethynyl-2′-deoxyuridine (EdU) staining *in vitro* and *in vivo

MEPM cells were applied an EdU solution (C0078S, Beyotime, China) to be treated for 4 h. 4% paraformaldehyde was used on the EdU-stained cells for 15 min. In vivo, EdU was solubilized in dimethyl sulfoxide (DMSO, 10 mg/kg, V900090, Sigma-Aldrich, Germany) and subsequently diluted with PBS. C57BL/6 mice received intraperitoneal injections of EdU at E13.5. After 2 h, cervical dislocation was utilized to the mice, and fetal litters were dissected to observe the development of the mouse palate.

### Cell counting kit-8 (CCK-8) assay

MEPM cells were treated with diluted CCK8 solution (CK04, Dojindo Laboratories, Japan) (CCK-8 solution: DMEM/F12 = 1:100). The microplate reader (10,822, Molecular Devices, United States) was used to perform the detection of each well at 450 nm.

### Apoptosis

MEPM cells were equally distributed throughout 6-well plates (3.5 × 10^5^ cells/well). The plate continued to incubate in 5% CO_2_ incubator (37 °C). Then, the cells were extracted by utilizing trypsin digestion, which without EDTA. After that, the MEPM single-cell suspension were stained with Apoptosis Assays Kit (C1062L, Beyotime, China). The cells were subsequently examined using flow cytometry (Accuri C6, Becton, United States).

### Immunofluorescence (IF)

MEPM cells were treated with 4% paraformaldehyde to preserve their structure, then exposed to Triton (0.25%, 10 min) to enhance permeability. Subsequently, the goat serum was used to seal for 1 h. After incubated the first and second antibody (Table S1), DAPI was used for nuclear staining.

### Assessment of glycolysis

The glucose uptake was measured by Glucose Uptake Colorimetric Assay kit (ab136955, Dojindo Laboratories, Japan). The measurement of lactate production was conducted utilizing the Lactate Assay Kit II (KTB1100, Abbkine, China) at a wavelength of 545/605 nm by microplate reader. The Glycolytic Rate was quantified utilizing the Seahorse XF Glycolytic Rate Assay Kit (103,344–100, Seahorse Bioscience, United States) following the kit's protocols. The examination was conducted with the Seahorse XFe 24 Extracellular Flux Analyzer (102,340–001, Seahorse Bioscience, United States).

### Statistical analysis

Statistical analyses were performed with GraphPad 9 (Prism, USA). Results were reported as means ± standard deviation. All data were normally distributed and variances were homogeneous by Shapiro–Wilk test, F test and Brown-Forsythe test. Unpaired two-tailed tests were applied when comparing two groups and one-way analysis of variance (ANOVA) was employed when comparing more than two groups. ANOVA followed by Tukey's Honestly significant difference (HSD) test. A p-value < 0.05 was considered statistically significant. All determinations were done in triplicate.

## Results

### The Pten inhibitor VO-OHpic could partially rescue the mouse embryonic CP induced by RA

**Fig. 1:**
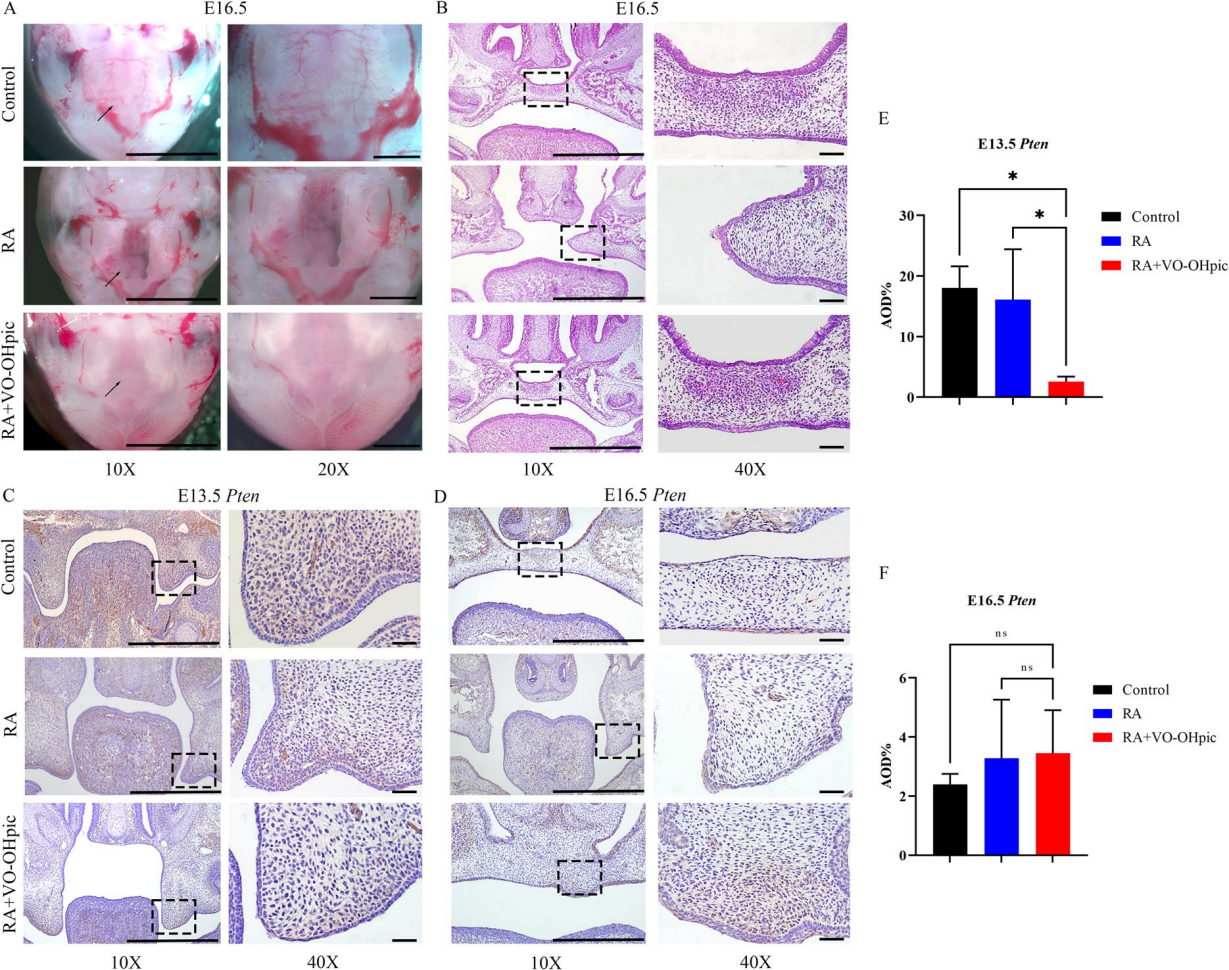
*Pten* inhibitor VO-OHpic partly rescued the mouse embryonic CP induced by RA. **(A)** Stereomicroscopy showing the development of the palatal tissues at E16.5 knockdown (10 × , magnification × 100, scale bar 100 μm; 20 × , magnification × 200, scale bar 50 μm). **(B)** H&E staining showing the development of the palatal tissues at E16.5 knockdown (10 × , magnification × 100, scale bar 100 μm; 40 × , magnification × 400, scale bar 20 μm). **(C, E)** IHC staining showing the expression of *Pten* at E13.5 and quantitative analysis. **(D, F)** IHC staining showing the expression of *Pten* at E16.5 and quantitative analysis (10 × , magnification × 100, scale bar 100 μm; 40 × , magnification × 400, scale bar 20 μm). AOD, average optical density. ^*^p < 0.05 compared with negative control group. ns, no significance

Firstly, we carried out a screening of VO-OHpic administration concentrations (10 μg/kg, 100 μg/kg, 1000 μg/kg) and observed the embryos at E16.5. As for the fetus, the results showed that the volume of embryos in RA group and RA + 10 μg/kg VO-OHpic group were smaller than control group and RA + 100 μg/kg VO-OHpic group, and RA + 1000 μg/kg VO-OHpic group could lead to stillbirth. Then to detect the palatal fusion in fetal mice, we observed the head of fetal mice by microscope. The fetal mice injected with 10 μg/kg VO-OHpic still had CP, and those injected with 1000 μg/kg VO-OHpic exhibited obvious malformations while in 100 μg/kg VO-OHpic group, the CP condition was rescued (Supplement Fig. 1). According to these results, we chose 100 μg/kg for subsequent experiment. The findings indicated that the control palatal shelves had fully made contact and stuck together at E16.5, but the anterior and posterior palatal shelves of embryos treated with RA were still not raised. Compared to the controls, the RA treatment resulted in a broader cleft phenotype, characterized by a significant gap in the secondary palate. While in RA + VO-OHpic group, it’s notably that palate shelves of the fetal mice had completely contacted and adhered with unobvious palatal folds compared with the wild type (43/51) (Fig. 1A-B). The results of IHC staining showed that the expression of *Pten* was decreased at E13.5 (Fig. 1C, E), but at E16.5 here was no significant difference among the three groups (Fig. 1D, F). According to the above, VO-OHpic partially saved mouse embryonic CP caused by RA.

### Pten knockdown promoted glycolysis by up-regulating and translocating glucose transporter type 1 (Glut1) in MEPM cells which subsequently increased MEPM cells proliferation

Then we wanted to investigate how *Pten* affected palate development. We firstly detected the expression pattern of *Pten* during mouse palate development by qRT-PCR, WB, and IHC from E12.5–15.5 which was the main formation time of the secondary palate. Our data indicated that the expression of *Pten* was higher in E13.5–14.5 and most of *Pten* protein was located in palatal mesenchyme (Supplement Fig. 2). Then the MEPM cells were chosen for subsequent experiments.

**Fig. 2:**
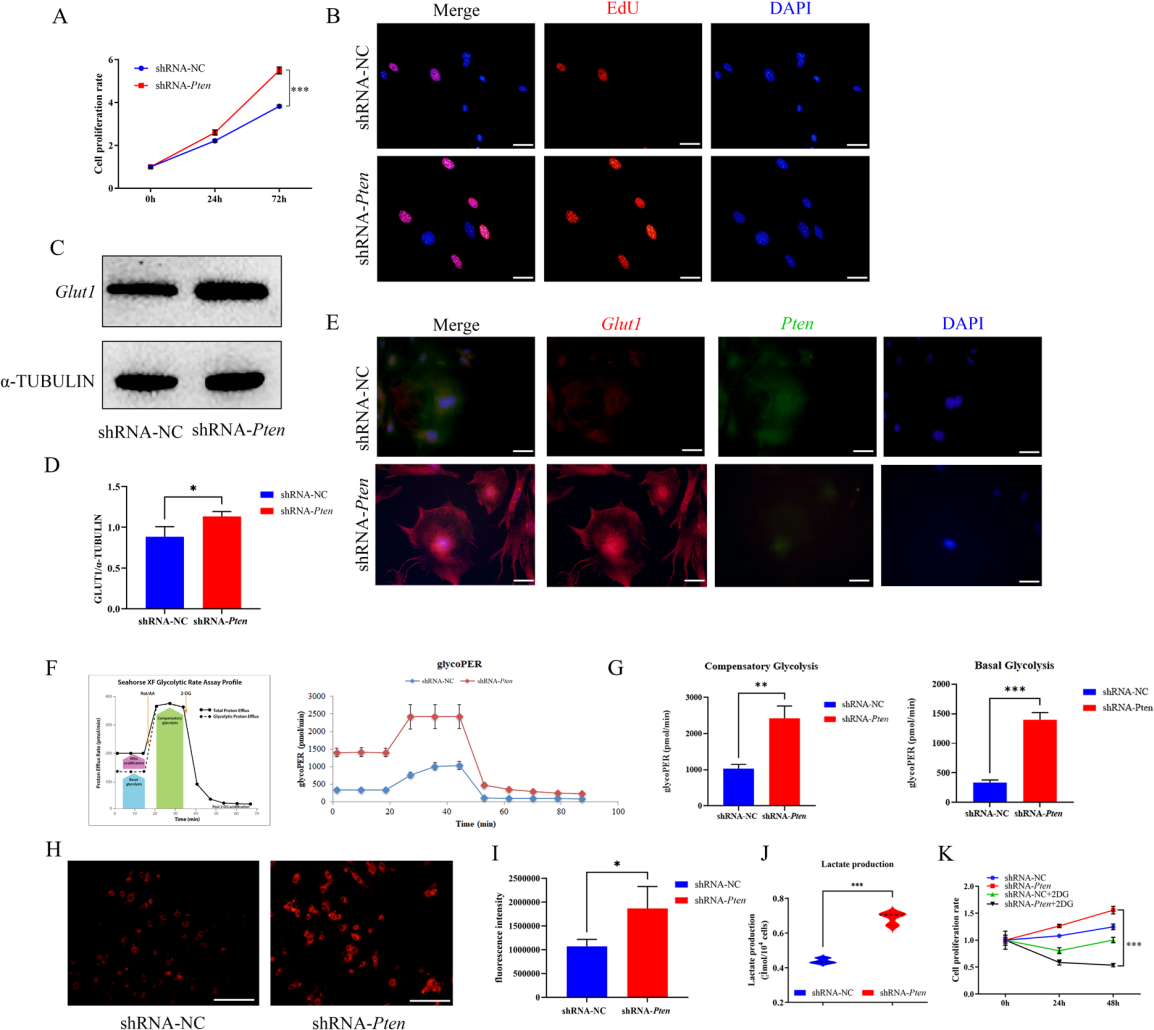
*Pten* knockdown promoted glycolysis function. **(A)** CCK-8 assay profile showing MEPM cells proliferation after *Pten* knockdown. **(B)** EdU staining showing MEPM cells proliferation after *Pten* knockdown (EdU, red fluorescent signals; DAPI, blue signals; magnification: × 400, scale bar 20 μm). **(C-D)** The protein level of *Glut1* were assessed with WB and quantitative analysis. **(E)** IF showing the *Glut1* plasma membrane translocation after *Pten* knockdown. (*Glut1*, red fluorescent signals; *Pten*, green fluorescent signals; DAPI, blue signals; magnification: × 400, scale bar 20 μm). **(F-G)** glycoPER were measured by Seahorse Bioscience XF24 analyzer in MEPM cells. **(H-I)** The ability of glucose uptake after *Pten* knockdown and statistical data (glucose uptake, red fluorescent signals; magnification: × 200, scale bar 50 μm). **(J)** The production of lactate after *Pten* knockdown. **(K)** 2-DG could inhibit the ability of proliferation in MEPM cells after *Pten* knockdown by CCK-8 assay. ^*^p < 0.05, ^**^p < 0.01, ^***^p < 0.001 compared with negative control group

Next we investigated how *Pten* regulated the biological characteristics of MEPM cells i*n vitro*. The results of qRT-PCR and WB showed that sequence1927 exhibited the best knockdown effect which was then selected in further experiments (Supplement Fig. 3A-C). CCK-8 results showed that the proliferation of MEPM cells was significantly increased compared with shRNA-NC group at 24–72 h after *Pten* knockdown (Fig. 2A). Results of the EdU staining also showed that red fluorescence, which represents proliferating MEPM cells, was significantly increased in shRNA-*Pten* group (Fig. 2B). However, the flow cytometry assay showed that the apoptosis ability of *Pte*n knockdown group did not change significantly (Supplement Fig. 3D-E).

Early studies on chicken and mouse embryos have confirmed that glycolysis predominates in early mammalian forebrain development (Fame et al. 2019). *Glut1* belongs to the glucose transporter family and controls the movement of glucose across the cell membrane (Deng et al. 2014). Glucose transmembrane transporters play a dual role as both the limiting factor in glycolysis and the initial step in glucose metabolism (Xiao et al. 2018). We then investigated the expression of *Glut1* after knocking down *Pten*. Our observations indicated a substantial increase in the protein levels of *Glut1* (Fig. 2C-D), and its plasma membrane translocation occurred after *Pten* knockdown (Fig. 2E). In order to further explore whether glycolysis affected MEPM cells through *Pten*, we investigated the glycolytic proton efflux rate (glycoPER) after *Pten* knockdown. Figure 2F-G showed that *Pten* knockdown up-regulated the glycolysis rate in MEPM cells, as evidenced by up-regulated basal glycolysis and compensatory glycolysis in MEPM cells. Next, we found that *Pten* knockdown enhanced glucose uptake capacity in MEPM cells. The red fluorescence represented the ability of cells to take up glucose, which significantly increased in shRNA-*Pten* group (Fig. 2H-I). At the same time, lactate production was also up-regulated in shRNA-*Pten* group (Fig. 2J). In order to verify the regulatory connection between glycolysis and proliferation, we applied the glycolysis inhibitor 2-Deoxy-D-glucose (2-DG, 50 μM, S4701, Selleck, United States), and Fig. 2K showed that 2-DG inhibited the proliferation of MEPM cells compared with non-2-DG groups. These results suggested *Pten* knockdown upregulated the expression and translocation of *Glut1*, which promoted the ability of glycolysis and then increased the proliferation of MEPM cells without changing their apoptosis.

### Pten knockdown activated AKT/GSK3β signaling pathways and SNX27-retromer complex which increased Glut1 and helped it translocate to the cell membrane to promote glycolysis

**Fig. 3:**
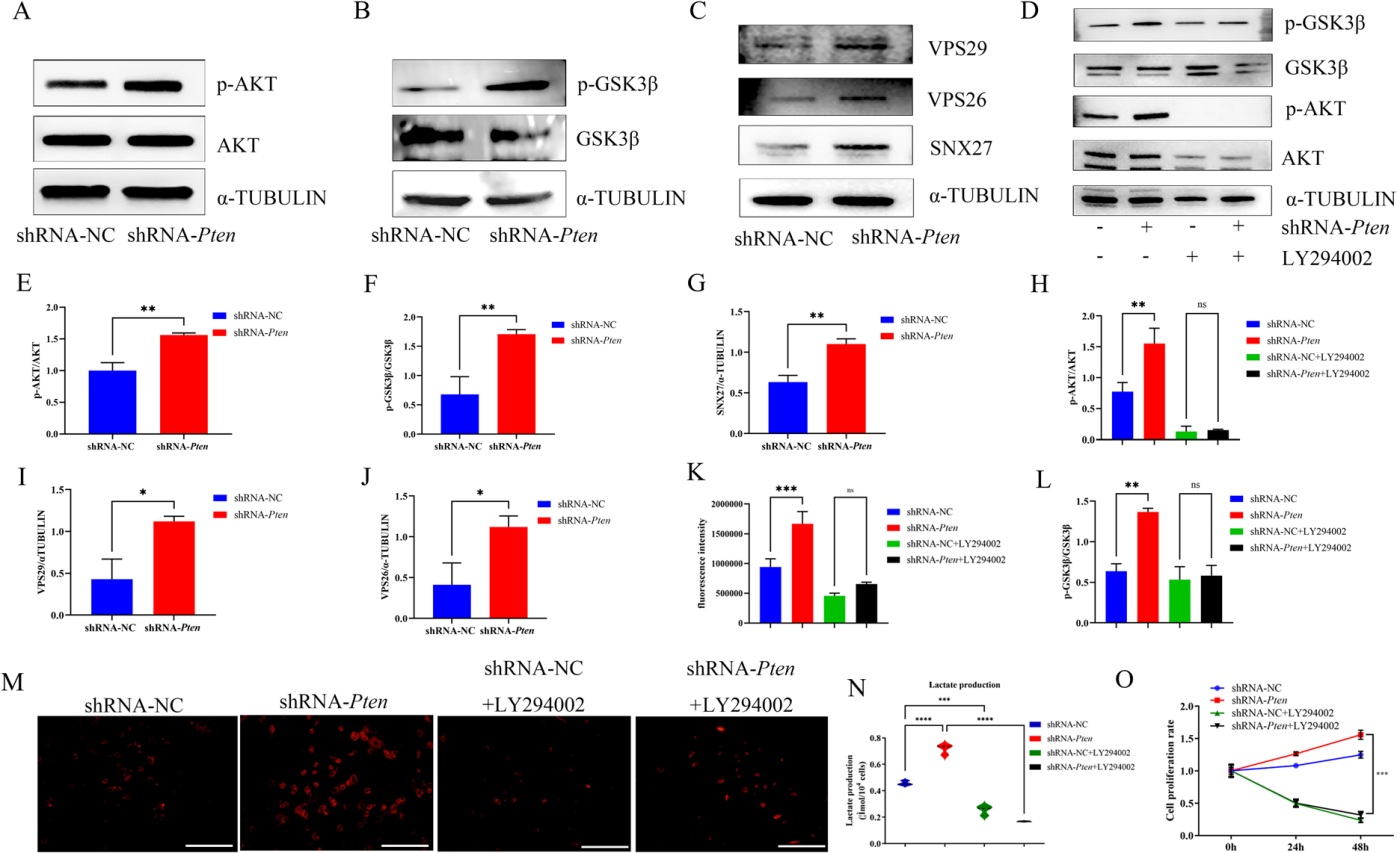
*Pten* knockdown promoted glycolysis function via AKT/GSK3β signaling pathways and SNX27-retromer complex **(A, E)** The protein levels of AKT and p-AKT were determined by WB and quantitative analysis. **(B, F)** The protein levels of GSK3β and p-GSK3β were determined by WB and quantitative analysis. **(C, G, I-J)** The protein levels of SNX27, VPS26 and VPS29 were determined by WB and quantitative analysis. **(D, H, L)** WB showing the protein levels of AKT, p-AKT, GSK3β and p-GSK3β after added LY294002 and quantitative analysis. **(K, M)** The ability of glucose uptake after added LY294002 and statistical data. (glucose uptake, red fluorescent signals; magnification: × 200, scale bar 50 μm). **(N)** The production of lactate after added LY294002. **(O)** CCK-8 assay profile showing MEPM cells proliferation after added LY294002. ^*^p < 0.05, ^**^p < 0.01, ^***^p < 0.001 and ^****^p < 0.0001 compared with negative control group. ns, no significance

Afterward, the underneath mechanisms of how *Pten* knockdown induced glycolysis were investigated. Since PI3K/AKT signaling is closely related to *Pten* (Bi et al. 2021) and was reported to interact with cell proliferation through glycolysis (Zhangyuan et al. 2020), we then focused on PI3K/AKT signaling. WB showed that the phosphorylation levels of AKT and glycogen synthase kinase 3β (GSK3β) were elevated in MEPM cells after *Pten* knockdown (Fig. 3A-B, E–F). SNX27 has been confirmed as a protein linked with *Pten*. *Pten* inhibits the recycling of *Glut1* from endosomes to the plasma membrane by interacting with the sorting nexins 27 (SNX27) -retromer complex (Shinde and Maddika 2017), retromer was mainly composed of vacuolar protein sorting (VPS) 26-VPS29-VPS35 trimers and Sorting Nexins (SNXs), a sorting linker protein (Liu et al. 2022), we investigated whether glycolysis and cell proliferation could be associated with SNX27-retromer VPS26 and VPS29. WB results showed that *Pten* knockdown increased the protein levels of SNX27, VPS26, and VPS29 (Fig. 3C, G, I, J).

**Fig. 4:**
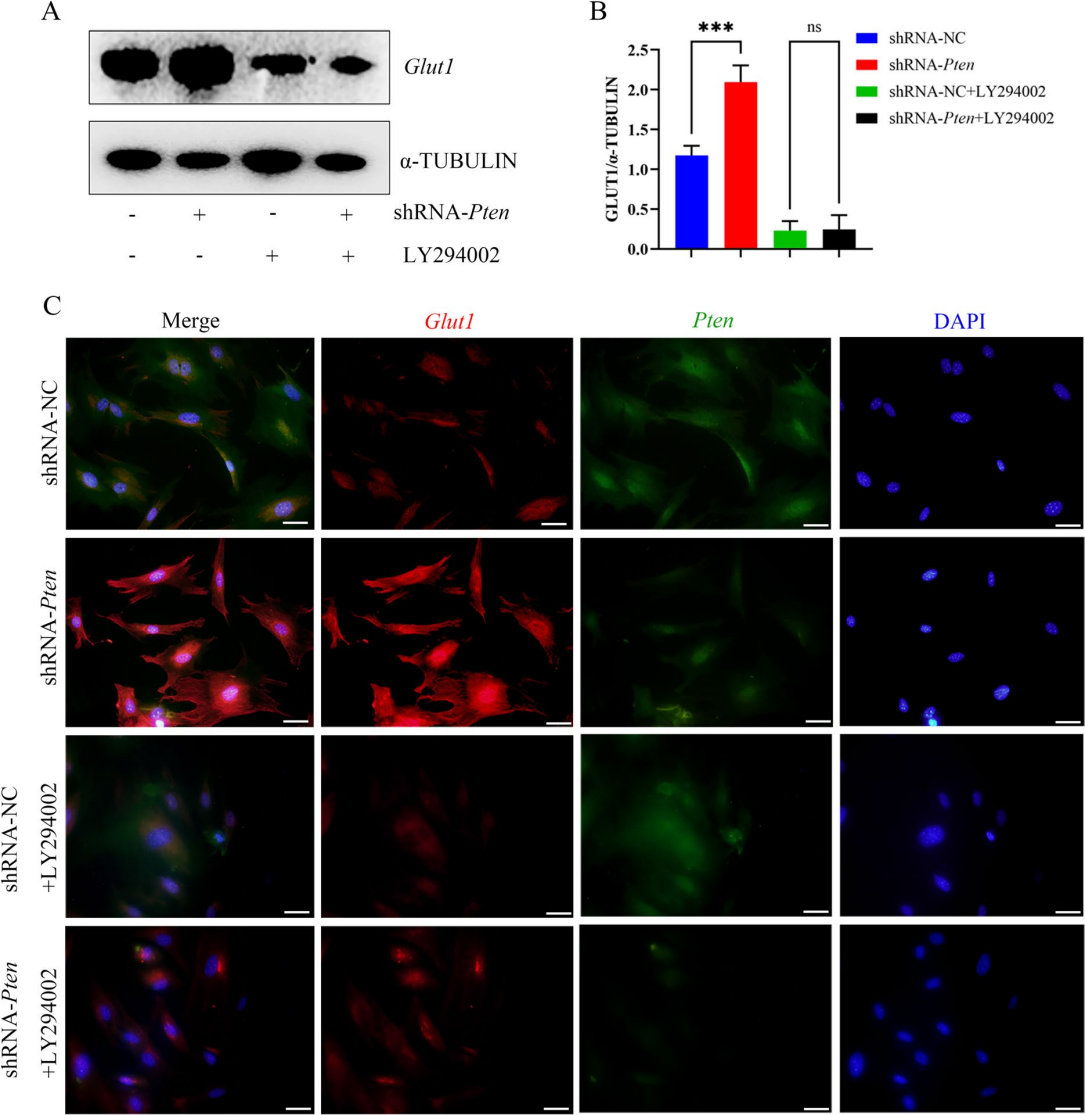
LY294002 inhibited the expression and translocation of *Glut1* **(A-B)** The protein levels of *Glut1* were assessed with WB after added LY294002 and quantitative analysis. **(C)** IF showing *Glut1* was located on cytoplasm after added LY294002 (*Glut1*, red fluorescent signals; *Pten*, green fluorescent signals; DAPI, blue signals; magnification: × 400, scale bar 20 μm). ^***^p < 0.001 compared with negative control group. ns, no significance

Then, we treated MEPM cells with PI3K/AKT pathway inhibitor LY294002 (10 μM, #9901, Cell Signaling Technology) (Chaikuad et al. 2014) for 6 h. WB analysis showed that the enhanced protein levels of p-AKT and p-GSK3β were inhibited by LY294002 (Fig. 3D, H, L). In addition, functions related to glycolysis were also inhibited, including glucose uptake (Fig. 3K, M) and lactate production (Fig. 3N). CCK-8 showed that LY294002 also inhibited the ability of proliferation in MEPM cells (Fig. 3O). WB also showed that the level of *Glut1* reduced (Fig. 4A-B) and IF showed that *Glut1* was located on cytoplasm after adding LY294002 (Fig. 4C).

### VO-OHpic promoted palatal proliferation and the expression of Glut1 by activating AKT and SNX27 pathways in fetal mice

**Fig. 5:**
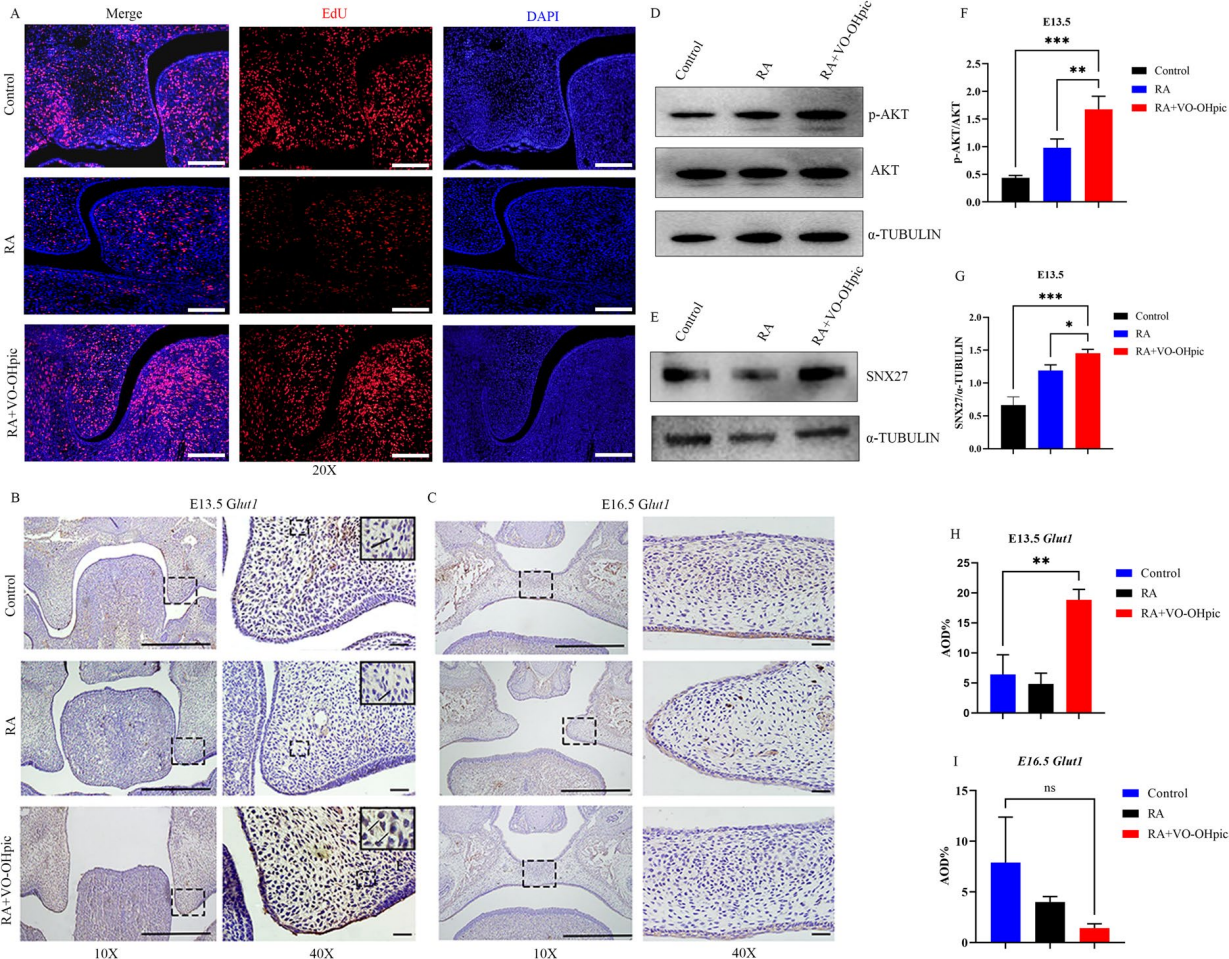
*Pten* inhibitor VO-OHpic facilitated palatal proliferation and the expression of *Glut1*, AKT and p-AKT and SNX27 in fetal mice. **(A)** EdU staining showing the ability of palatal tissues at E13.5. (EdU, red fluorescent signals; DAPI, blue signals; magnification: × 200, scale bar 50 μm). **(B, H)** IHC showing the expression of *Glut1* at E13.5 and quantitative analysis. **(C, I)** IHC showing the expression of *Glut1* at E16.5 and quantitative analysis. (10 × , magnification × 100, scale bar 100 μm; 40 × , magnification × 400, scale bar 20 μm). **(D)** The protein levels of AKT and p-AKT in the palatal tissue of E13.5 were determined by WB. **(E)** The protein levels of SNX27 in the palatal tissue of E13.5 were determined by WB. **(F, G)** Quantification of protein levels. ^*^p < 0.05, ^**^p < *0.01* and ^***^p < *0.001* compared with negative control group. ns, no significance

Then the proliferative capacity of mouse palatal shelves and the expression of *Glut1* were examined by EdU staining and IHC, respectively. Initially, we established the optimal concentration of EdU required for labeling whole mouse embryos. The fluorescence staining findings revealed a substantial decrease in the number of proliferating cells labeled in the RA group compared to the control group. Conversely, the number of proliferating cells labeled in the VO-OHpic group showed a considerable increase (Fig. 5A). IHC data revealed that the levels of *Glut1* was significantly higher in the palatal tissues of E13.5 fetal mouse compared with the Control and RA groups (Fig. 5B, H), but there was no discernible changing in the palatal tissues of E16.5 among three groups (Fig. 5C, I) which was consistent with *Pten* expression pattern. Next, we investigated the levels of AKT and SNX27 pathways and the phenotypes in our rescue models in vivo. Consistent with in vitro results, western blots revealed that the expression of p-AKT and SNX27 were up-regulated in the palatal tissue of E13.5 fetal mice (Fig. 5D-G).

**Fig. 6:**
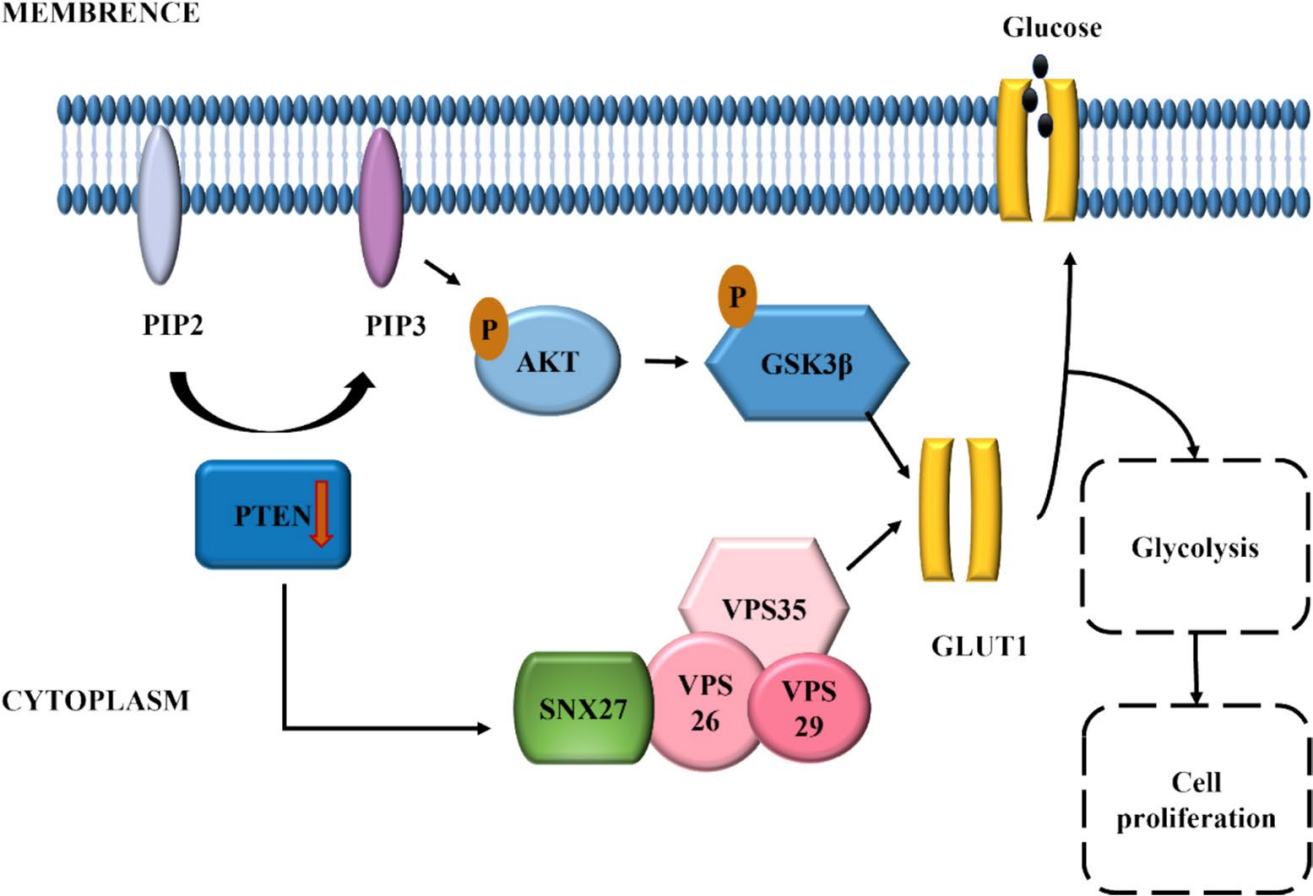
*Pten* knockdown activated AKT/GSK3β and SNX27-retromer complex signaling pathways and promoted glucose transport through up-regulated *Glut1*, thereby facilitated glycolysis in palatal mesenchyme and then promoted the function of MEPM proliferation

Overall, combined with the results in vitro and in vivo, *Pten* knockdown firstly activated classical AKT/GSK3β signaling pathways and non-classical SNX27-retromer complex pathways, then promoted glycolysis by increasing *Glut1* expression and helping it translocate to the cell membrane, facilitating the ability of MEPM cells proliferation which eventually reversed the occurrence of CP (Fig. 6).

## Discussion

The palatogenesis of mammalian is a tightly controlled process of shaping and development (Bush and Jiang 2012). Among them, proliferation is a significant factor in the formation of the palate (Jin et al. 2018; Logan et al. 2020; Siismets and Hatch 2020). As the common mammalian model on palate development, murine palatogenesis originates from E11.5, and E13.5 is a critical time point for cell proliferation during palatal outgrowth and for palatal shelves elevation (Peng et al. 2020; Yoshioka et al. 2021). The previous research findings of our group also confirmed that palatal shelves at E13.5 tended to proliferate (Peng et al. 2023). In our study, it was detected that *Pten* knockdown in E13.5 MEPM cells increased MEPM cells proliferation in vitro (Fig. 2A, B). Therefore, in order to better explore the issue of how *Pten* affects palatal development by regulating the proliferative capacity of the palate, we chose to inject VO-OHpic at this stage. And at E16.5, the elevated palatal shelves continuously grow horizontally toward each other and fuse when the early palate development finishes (Suzuki et al. 2018) and was chosen as the time point for CP investigation in many studies (Wang et al. 2017; Zhang et al. 2023), so we chose E16.5 to observe the situation of palatal fusion. RA is frequently employed to produce CP in mice, one of the main mechanisms of RA-induced CP was RA suppressed the proliferation of MEPM cells (Zhong et al. 2020). As *Pten*^fl/fl^; *Wnt1*Cre2 conditional knockout mice displayed complete perinatal mortality, characterized by larger heads and excessive development of palatal tissues (Yang et al. 2018), but it’s difficult to study the role of *Pten* in CP etiology. In our in vivo results, RA was used to model a CP and then *Pten* inhibitor VO-OHpic was applied to inhibit *Pten* expression in the fetal mice. It was surprising that CP induced by RA in mice was partially rescued by *Pten* inhibition. Early results also found that *Pten* acted a key role in the process of craniofacial development (Yang et al. 2018), but the mechanism is not clear.

In order to investigate the underneath mechanism, we knocked down the expression of *Pten* in MEPM cells. In our research, EdU and CCK-8 showed that *Pten* deficiency promoted the ability of proliferation in MEPM cells and the glycolytic capacity was also up-regulated, which was measured by seahorse, glucose uptake capacity, and lactate amount. As a crucial function of early embryonic development, glycolysis exhibited an important role in the regulation of posterior elongated tail buds and cells of the posterior Presomitic mesoderm (PSM) (Oginuma et al. 2020), which was also closely related to the ability of cell proliferation (Koprivica et al. 2020; Lunt and Vander Heiden 2011). But the relationship between proliferation and glycolysis in MEPM cells is still unknown. To investigate the relationship between glycolysis and proliferation, we added the glycolysis inhibitor 2-DG and found that the proliferation ability of MEPM cells was inhibited. These results suggested *Pten* knockdown could promote glycolysis which then increased the proliferation of MEPM cells. As the main reason for increased glycolytic capacity was increased glucose intake in MEPM cells, we then detected *Glut1*, the key glucose transporter, was significantly upregulated after *Pten* knockdown. Glucose is the main energy supplier for human cells, and mammalian cells convert glucose into pyruvate and ATP for cellular use mainly through glycolysis (Tang 2020). Glucose relied on membrane transporter proteins to mediate transmembrane diffusion into cells, of which *Glut1* was the main glucose transmembrane transporter protein and was ubiquitously presented in human tissues and organs (Joost et al. 2002). To keep malignant cell proliferation going strong, it was important to keep glucose transmembrane transport efficient, since excessively active aerobic glycolysis used up a lot of glucose in tumors (Zhang et al. 2021). Studies also reported that in tumor cells, *Pten* regulated *Glut1* expression through PI3K-dependent and independent pathways, thereby affecting the metabolic state of tumor cells. The dependent pathway, which was through the classical PI3K/AKT signaling pathway, was mainly manifested by *Pten* deletion and could promote glucose uptake by phosphorylating the PI3K/AKT signaling pathway, causing extensive expression of *Glut1* at the plasma membrane (Phadngam et al. 2016; Tang et al. 2020). Our results demonstrated that PI3K/AKT pathway inhibitor LY294002 downregulated the expression of *Glut1* whenever *Pten* was knocked down which meant PI3K/AKT pathway was downstream of *Pten* to regulate *Glut1* in MEPM cells.

Furthermore, the silencing of *Pten* served as a pivotal element in inhibiting the PI3K/AKT signaling pathway, this inhibition resulted in an upsurge in neuronal proliferation and differentiation by activating the PI3K/AKT/GSK3β pathway (Song et al. 2018). According to reports, there was a correlation between GSK3β and the glycolysis capacity in HepG2 and Hep 3B cells (Zhong et al. 2020). The phosphorylation state of Ser9 impacted the activity of GSK3β. Akt1 was a crucial protein kinase that control the phosphorylation of GSK3β at Ser9, resulting in the reduction of GSK3β activity through the increased of GSK3β phosphorylation at Ser9 (Zhong et al. 2020). Previous studies have mostly demonstrated the regulatory function of GSK3β on glycolysis in cancer. In our results, GSK3β was also found to affect the regulation of glycolytic function during palate development which was not reported before.

On the other hand, the SNX27 protein, which possessed the PDZ domain, promoted the transportation of transmembrane proteins from endosomes back to the plasma membrane. It did this by connecting the recognition of specific cargo with the transport mechanism mediated by retromer (Steinberg et al. 2013). Research work has demonstrated that *Pten* could govern glucose transport via SNX27-retromer pathway by preventing *Glut1* accumulation at the plasma membrane (Shinde and Maddika 2017). Also, SNX27 demonstrated a key role in maintaining glucose supply and glycolysis, which was related to *Glut1* (Zhang et al. 2022). In our research, we determined that *Pten* knockdown could promote the expression of SNX27. At the same time, the levels of the retromer complex VPS26 and VPS29 were also up-regulated after *Pten* knockdown. All of these results showed that *Pten* knockdown facilitated glycolysis and *Glut1* transposition was related to the up-regulated of SNX-27 retromer. Next, we analyzed the data in mice embryo palate shelves to verify the results in vitro. It was investigated that blocking *Pten* also promoted the proliferative capacity and increased *Glut1* enhancement and translocation to the cell membrane by activating AKT and SNX27 pathways in the mesenchyme of palatal shelves, which were consisted with the results in vitro. And interestingly after the palate development was due at E16.5, the expressions of *Pten* and *Glut1* were down-regulated and had no difference among the three groups which displayed *Pten*-regulated palate development in a space–time specificity manner. Binds our results and the previous report (Yang et al. 2018), we speculated that *Pten* might act as a "trigger point" and controlled the rate of palatal development by regulating glycolysis via AKT/GSK3β signaling pathway and SNX27-retromer complex.

Currently, genetic screening played an important role in the detection of genetic diseases (Garzon and Wong 2009). Many genetic developmental diseases could be effectively diagnosed by gene chips, such as hereditary spastic paraplegias, hereditary hearing loss and so on (Küçük Kurtulgan et al. 2019; Luo et al. 2013). Due to the complex pathogenesis of CP, it’s difficult to obtain effective hub genes until now. As interceptive treatment is proved to be an effective treatment in many diseases such as HIV exposer and harmful environment to pregnant mother is one of the major etiology of CP, interceptive treatment may be another direction to prevent CP occurring. Consequently, in the subsequent research, we will conduct a more in-depth elucidation of the action mechanism of VO-OHpic on CP. To be specific, we will perform comprehensive analyses on the palatal development of fetal mice at various developmental stages, placental mass, and the size and weight of the offspring body. Through these investigations, we aim to provide more robust evidence for the effects of VO-OHpic in the treatment of CP. Simultaneously, we will explore the influence of VO-OHpic on the physiological functions of various organs in both the maternal and offspring organisms. The objective is to determine whether VO-OHpic induces drug-related toxicity in the mother and offspring. Additionally, we will employ multi-omics analysis to conduct a profound exploration of the interaction mechanism between *Pten* and SNX27-retromer, thereby furnishing more substantial evidence for the non- classical pathways of *Pten*.

Our research provides a novel target in protective or interceptive treatment for CP due to proliferation abnormalities such as blocking *Pten*, which might offer some significance esp. for pregnant mothers who expose in harmful environment inducing CP in the early development of palate in clinical practice.

## Conclusions

The present study elucidated the role of glycolysis during palate development which was not reported before and investigated the crosstalk between *Pten* and glycolysis. These findings provide novel insights into the etiology of CP with the role of metabolism during palate development and present a new direction for exploring the interceptive treatment of CP.

## Supplementary Information

Below is the link to the electronic supplementary material.

Supplementary file1 (DOCX 2912 KB)

## Data Availability

No datasets were generated or analysed during the current study.
