# Supplementary material for: Glycolysis regulates palatal mesenchyme proliferation through Pten-Glut1 axis via Pten classical and non-classical pathways
Source: Cell Biol Toxicol. 2025 Feb 27;41(1):53. doi: 10.1007/s10565-025-10000-2 (PMC11868302; doi:10.1007/s10565-025-10000-2)
Supplement: Supplementary file 1 — Supplementary file1 (DOCX 2912 KB) [file 10565_2025_10000_MOESM1_ESM.docx]

**Glycolysis regulates palatal mesenchyme proliferation through *Pten-Glut1* axis *via Pten* classical and non-classical pathways**

Yijia Wang^1^, Xia Peng^1^, Xiaotong Wang^1^, Jing Chen^1^, Xiaoyu Zheng^1^, Xige Zhao^1^, Cui Guo^2^, Juan Du^1,2^*

**Appendix**

**Appendix Figure**

**
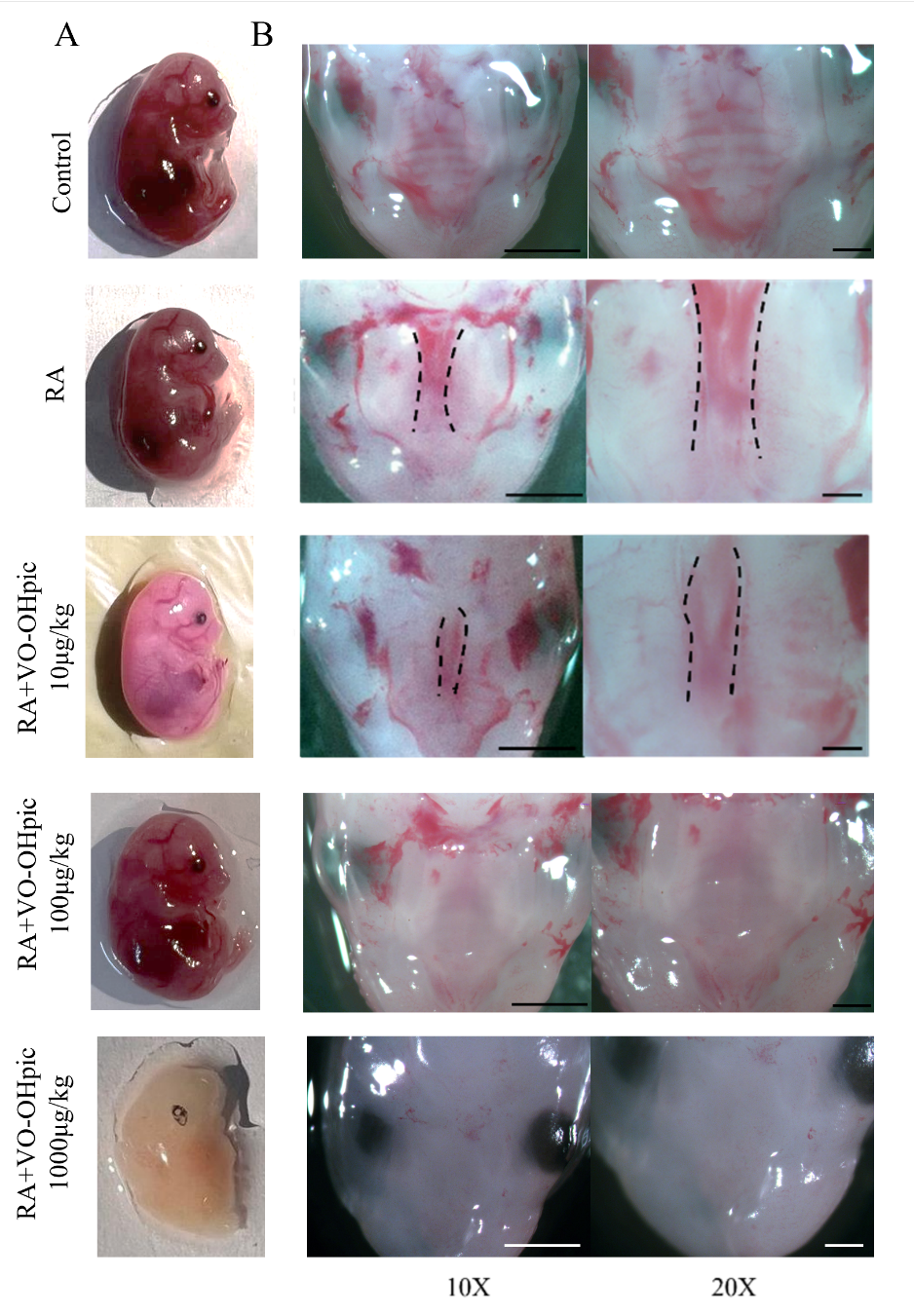
**

**Fig. S1** Screening of VO-OHpic administration concentrations. **(A)** Fetus. **(B)** Palatal shlves (10×, magnification ×10, scale bar 100 mm; 20×, magnification ×20, scale bar 50 mm).

**
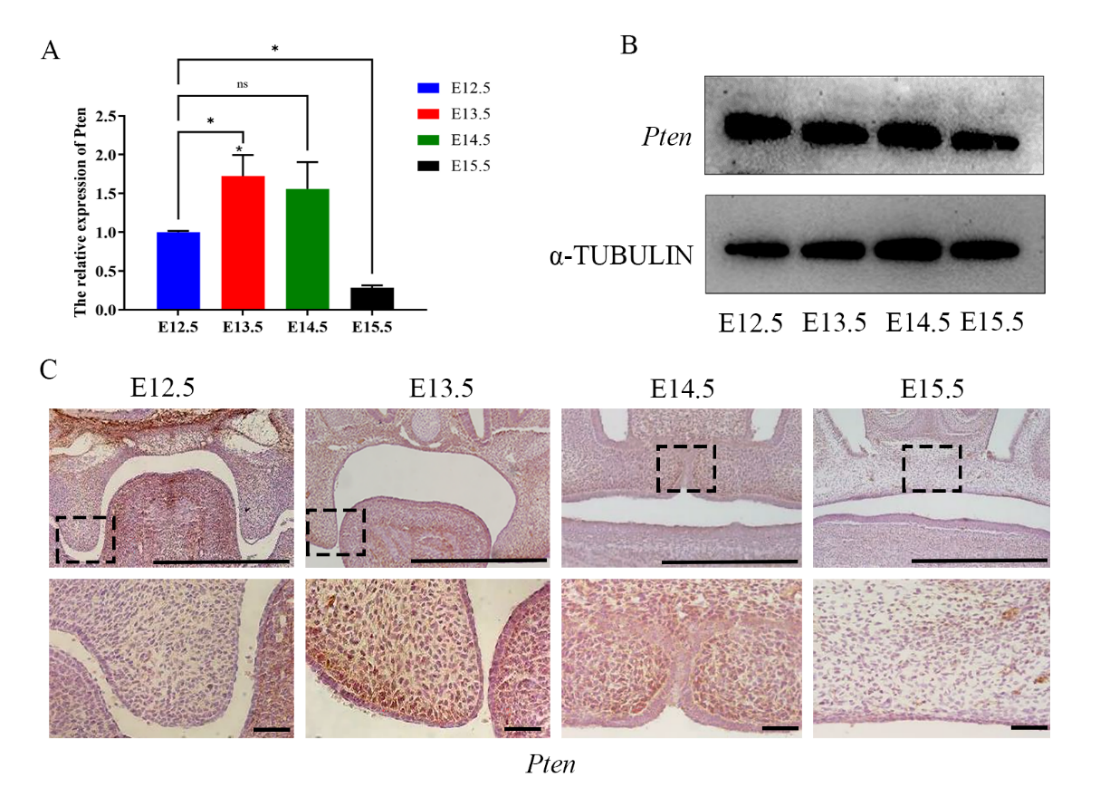
**

**Fig. S2** The expression of *Pten* in palatal mesenchyme was spatiotemporal specific. **(A)** The expression of *Pten* in palatal tissue from E12.5-15.5 by qRT-PCR. **(B)** The protein level of *Pten* in palatal tissue from E12.5-15.5 by WB. **(C)** IHC showing the expression of *Pten* from E12.5-15.5. (10×, magnification ×100, scale bar 100 μm; 40×, magnification ×400, scale bar 20 μm). ^*^*p*＜0.05 compared with negative control group. ns, no significance.

**
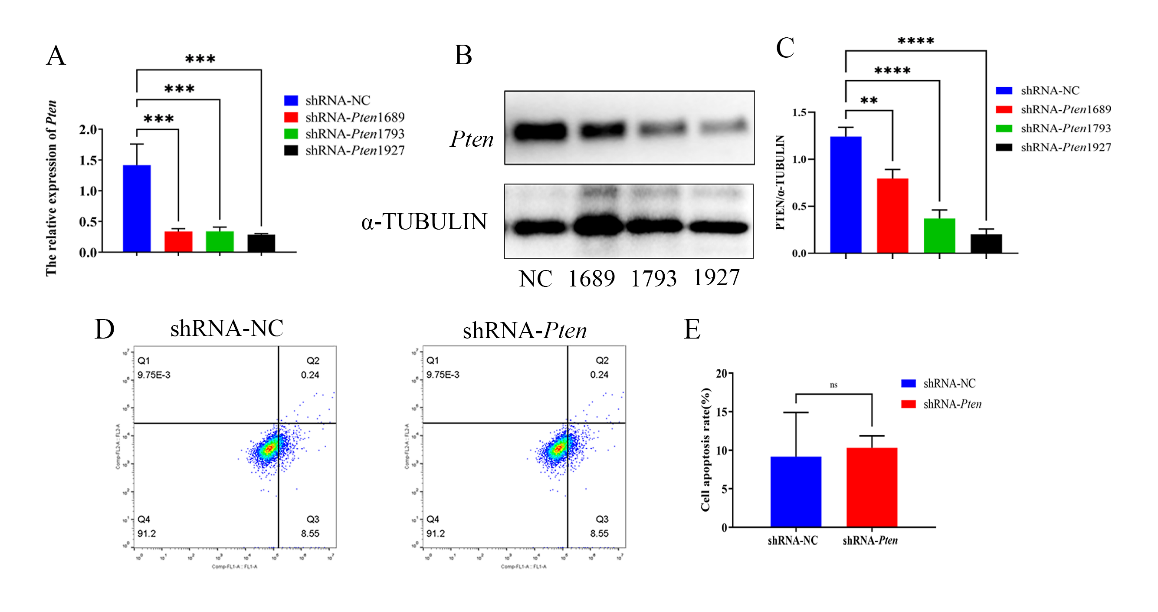
**

**Fig. S3** *Pten* knockdown did not change its apoptosis. **(A)** MEPM cells were transfected with shRNA and total RNA was extracted. The expression of three *Pten* isoforms was assessed with qRT-PCR. **(B-C)** MEPM cells were transfected with shRNA and total protein was extracted. The protein levels of three *Pten* isoforms were assessed with WB and quantitative analysis. **(D-E)** Representative images of flow cytometry analysis after *Pten* knockdown and statistical data of cell apoptosis. ^**^*p* < 0.01, ^***^*p* < 0.001 and ^****^*p* < 0.0001 compared with negative control group. ns, no significance.

**Appendix Tables**

**Table S1 Antibodies for IHC, IF and western blots**

| Antibody | Product code |
| --- | --- |
| PTEN | #9552, Cell Signaling Technology |
| GLUT1 | 21829-1-AP, Proteintech |
| α-TUBULIN | AC007, ABclonal |
| AKT | #4685, Cell Signaling Technology |
| p-AKT | #4060, Cell Signaling Technology |
| GSK3β | 22104-1-AP, Proteintech, |
| p-GSK3β | #5558, Cell Signaling Technology |
| SNX27 | ab241128, Abcam |
| VPS26 | sc-390304, Santa Cruz |
| VPS29 | A13098, ABclonal |
| VPS35 | 10236-1-AP, Proteintech |

**Table S2 Sequences for small-hairpin RNA**

| RNA oligo | Sequence (5′- 3′) |
| --- | --- |
| Negative control | TTCTCCGAACGTGTCACGT |
| sh*Pten*-1689 | GGGTAAATACGTTCTTCATAC |
| sh*Pten*-1793 | GCAGATAATGACAAGGAGTAT |
| sh*Pten*-1927 | GCCATCAAATCCAGAGGCTAG |

**Table S3 Primer sequences for qRT-PCR**

| Gene | Forward primer 5’-3’ | | Reverse primer 5’-3’ | |
| --- | --- | --- | --- | --- |
| *β-actin* | GTGACGTTGACATCCGTAAAGA | | | GCCGGACTCATCGTACTCC |
| *Pten* | ACCCGTGGCACTGCTGTT | GATATCACCACACAGGCAATG | | |

**Data access**

The study data is available in the manuscript and appendix submission.
